# Construction and Verification of a Mathematical Model of Storage Time With Respiratory Heat Transfer

**DOI:** 10.1155/ijfo/4258197

**Published:** 2026-07-08

**Authors:** Liying Duan, Haozhe Liu, Zhiqiang Fu, Liqiang Huang, Yan Wang

**Affiliations:** ^1^ Hebei Key Laboratory of Intelligence Equipment Digitalized Design and Process Simulation, Tangshan University, Tangshan, Hebei, China, tsc.edu.cn; ^2^ Department of Mechanical and Energy Engineering, Southern University of Science and Technology, Shenzhen, Guangdong, China, sustc.edu.cn; ^3^ School of Light Industry Science and Engineering, Tianjin University of Science and Technology, Tianjin, China, tust.edu.cn; ^4^ College of Mechanical Engineering, Tianjin University of Science and Technology, Tianjin, China, tust.edu.cn

**Keywords:** cold chain container, respiratory heat model, storage time, theoretical model

## Abstract

Cold chain containers are extensively applied to the fruit transport sector, and the storage time is a key performance indicator. The correlation between fruit respiration and storage longevity has not been fully elucidated. A new theoretical model is constructed by coupling conduction–convection–radiation–respiratory heat transfer, taking lemon, mango, and orange as examples. As for the cold chain container, heat transfer was constructed by shape factors, gravity–temperature difference coupling, and the radiation network method. As for fruit, the nonlinear model is used to fit the respiratory intensity at different temperatures. Theoretical predictions were compared with experimental data and simulation outcomes, which demonstrate the ability of the proposed theoretical model to serve as a substitute for simulation models in predicting experimental outcomes. Compared with the theoretical model without respiratory heat, the high prediction accuracy for the experimental results was obtained by the new theoretical model with respiratory heat. These findings reveal how fruit respiration impacts storage time in cold chain units, thereby guiding design optimization to minimize energy loss.

## 1. Introduction

Maintaining a low‐temperature environment is crucial for preserving the quality of fresh fruits during transportation [1]. In cold chain transportation, cold chain containers can maintain a stable low‐temperature environment within their enclosed interior [2] and address the “last kilometer cold chain break” issue [3]. Owing to these advantages, cold chain containers are widely employed in fruit transportation [4, 5]. For cold chain containers, storage time is regarded as a critical performance metric that influences the selection of transportation distances [6, 7]. In this study, storage time is defined as the duration for which the representative fruit temperature inside the container remains below the maximum permissible transportation temperature (10°C) under a given ambient temperature.

Currently, research on storage time is centered around three main approaches: experimental testing [8, 9], CFD simulation [10, 11], and theoretical modeling. Experimental methods enable direct and accurate measurement of storage time, yet they are constrained by intensive requirements for time, labor, and resources [12, 13]. While CFD simulations can address some of these constraints by providing detailed flow and temperature fields [14, 15], such simulations suffer from long runtimes, prohibitive computational costs, and strong dependence on hardware performance. As a more efficient alternative, theoretical thermal models offer comparable predictive capacity without the heavy computational and hardware requirements of CFD simulations. Nevertheless, existing theoretical models have largely focused on conductive, convective, and radiative heat transfer of the container itself [14–16], neglecting the influence of the internal fresh fruit cargo—particularly its respiratory heat release on the storage time. This omission in thermal models can lead to significant underestimations of internal heat loads, resulting in inaccurate storage performance predictions and potential economic losses. Consequently, integrating respiratory heat generation into the mathematical modeling framework is essential to improve the prediction accuracy of thermal performance for cold chain containers.

As a continuous metabolic process in fresh fruits, fruit respiration releases heat that acts as a nonnegligible internal heat load inside the cold chain container, directly impacting the accuracy of thermal performance predictions [17, 18]. The magnitude of this heat release is governed by respiratory intensity [19], whose transient magnitude depends primarily on whether the fruit exhibits a climacteric mechanism. Therefore, mango (a typical climacteric fruit exhibiting a respiratory surge) and lemon and orange (nonclimacteric fruits with relatively stable metabolic rates) were selected as samples.

To quantify the dynamic respiratory heat release of the three selected fruits and integrate it into the cold chain container thermal model, it is necessary to select an applicable respiration rate prediction model. Lee et al. [20] proposed a fruit respiration model based on enzyme kinetics, with a specific focus on uncompetitive inhibition. Kandasamy [17] improved this model by considering the impact of ambient CO_2_ concentration on respiratory intensity. However, this model proved too complex for theoretical coupling, with too many temperature‐dependent parameters [21]. To simplify, Bhande et al. [22] applied a nonlinear model, which achieves superior fitting accuracy with experimental data compared to the enzyme kinetics–based model. Ravindra and Goswami [23] found that this nonlinear model accurately described mango respiration. Accordingly, this nonlinear respiration model is selected to construct the respiration rate prediction models for the three selected fruit samples in this study.

In this study, we first derived the temperature‐dependent parameters of the nonlinear respiration model selected to accurately quantify the dynamic internal metabolic heat source inside the container. We established a novel theoretical model coupled with conduction, convection, radiation, and respiratory heat transfer mechanisms based on this quantified heat load. The theoretical model is thoroughly verified against both experimental and CFD simulation results. Furthermore, the role of respiratory heat in governing the storage time is quantitatively analyzed by comparing the proposed model with a baseline theoretical model lacking the respiratory heat component. The findings are expected to provide a computationally efficient theoretical tool for the optimized design and energy‐efficient operation of refrigerated containers, offering guidance for minimizing both logistic losses and energy consumption.

## 2. Model Description

### 2.1. Theoretical Model

To predict the storage time (*Δ* *t*) of the cold chain container, a transient energy balance equation for the internal fruit system was first established. Based on the first law of thermodynamics, the fruit is treated as a control volume where the change in internal energy equals the sum of net heat flux and internal heat sources [14, 15, 24]:

(1) ΔEsthx=Einhx−Eouthx+Egice,

where *Δ* *E*_sthx_ is the change in thermal and mechanical energy of fruit during the storage period (Joules), *E*_inhx_ is the energy input into fruit from the external environment (Joules), *E*_outhx_ is the energy output from fruit to the external environment (Joules), and *E*_gice_ is the energy of ice (Joules).

In Equation (1), the internal energy change of the system (*Δ* *E*_sthx_) consists of two components: the sensible heat required to reduce the fruit temperature from ambient to the optimal storage level and the metabolic heat continuously generated by fruit respiration. Consequently, *Δ* *E*_sthx_ is expanded as follows:

(2) ΔEsthx=chxMhxTbesthx−Tinitialhx+Qr,

where *c*_hx_ is the specific heat of fruit (J.kg^−1^·°C^−1^), *M*_hx_ is the mass of fruit (kilograms), *T*_besthx_ is the best transportation temperature of fruit (°C), *T*_initialhx_ is the initial temperature of fruit (°C), and *Q*_r_ is the respiratory heat of fruit (Joules).

**Figure 1:**
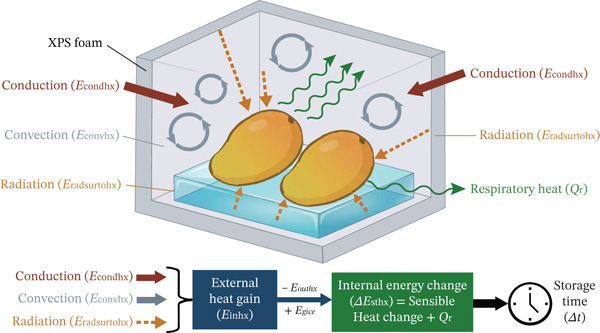
Schematic diagram of the coupled heat transfer mechanisms and energy balance within the cold chain container. The diagram reveals that the internal energy change (*Δ* *E*_sthx_) can be expressed by two equivalent equations: the net external energy exchange combined with the refrigerant phase change energy (*E*_inhx_ − *E*_outhx_ + *E*_gice_) and the sum of the fruit′s sensible heat variation and metabolic respiratory heat (Equation 2). The storage time (*Δ* *t*) is derived by simultaneously solving these two equations.

The coupling logic of these aforementioned heat transfer mechanisms, along with the detailed composition of the internal energy change (*Δ* *E*_sthx_), is intuitively depicted in Figure 1.

Microbial respiration is aerobic respiration [17, 25]:

(3) C6H12O6+662882O2⟶CO2+H2O+KJ.

As for respiratory heat, only 55% energy of aerobic respiration is used for heat transfer. Therefore, the heat of respiration is as follows [17, 25]:

(4) Qr=0.552282×MhxRco2Δt6,

where $R_{\text{c}{\text{o}}_{2}}$ is the respiration rate of fruit (m^3^·kg^−1^.h^−1^) and *Δ* *t* is the storage time of the theoretical model (minutes).

In Equation (4), respiration intensity is related to the change of CO_2_ concentration in the cold chain container [17, 25]:

(5) RCO2=VhxdCCO2MhxdΔt,

where *V*_hx_ is the volume of fruit (cubic meters), $C_{\text{C}{\text{O}}_{2}}$ is the specific heat of fruit (J.kg^−1^·°C^−1^), *M*_hx_ is the mass of fruit (kilograms), and *T*_besthx_ is the best transportation temperature of fruit (°C).

As for fruit, the nonlinear model is used to fit the respiratory intensity. The relationship between the CO_2_ concentration and time is as follows [22]:

(6) CCO2=ΔtaΔt+b,

where *a* and *b* are the parameters of the respiratory rate.

By differentiating this equation, the rate of CO_2_ concentration change, namely, the respiration intensity, is obtained [22]:

(7) dCCO2dΔt=−aΔtaΔt+b2+1aΔt+b=baΔt+b2.

Substituting Equation (7) into Equations (5) and (4), the time‐coupled expression for respiratory heat is derived, completing the modeling of the internal heat source term within the internal energy change (*Δ* *E*_sthx_) [22]:

(8) Qr=0.552282××VhxbΔt6aΔt+b2.

Next, the external heat transfer term (*E*_inhx_) in Equation (1) is modeled. The energy transferred from the ambient environment to the fruit is the superposition of three mechanisms: conduction, convection, and radiation [24]:

(9) Einhx=Econdhx+Econvhx+Eradsurtohx,

where *E*_condhx_ is the conductive heat transfer from the surrounding environment to the fruit (Joules), *E*_convhx_ is the convective heat transfer from the surrounding environment to the fruit (Joules), and *E*_radsurtohx_ is the radiative heat transfer from the surrounding environment to the fruit (Joules).

As for *E*_condhx_, it is calculated using the shape factor method, with detailed derivations and intermediate steps provided in [14–16]. The final formula for *E*_condhx_ in the cold chain container is as follows:

(10) Econdhx=ksΔTΔt=80.150.54×kXPS+PkXPS+∑Ain−∑Aice−∑AhxxkXPS+∑Ahx1/x/kXPS+Hair/kair+Hhx/khxTsur−TbesthxΔt+∑Aice1/Hice/2kice+Hhx/khxTs−TbesthxΔt+∑Aice1/Hice/2kice+x/kXPSTsur−TsΔt,

where *k*_XPS_, *k*_air_, *k*_ice_, and *k*_hx_ are the thermal conductivities of XPS, air, ice, and fruits (W.m^−1^·°C^−1^), *H*_ice_, *H*_hx_, and *H*_air_ are the heights of ice, fruit, and air in the cold chain container (meters), *A*_in_ is the internal surface area of cold chain container (square meters), *A*_ice_ and *A*_hx_ are areas of ice and fruit (square meters), *T*_sur_ is the ambient temperature (°C), *T*_s_ is the phase change temperature (°C), and *x* is the thickness of cold chain container (meters).

For the convection term (*E*_convhx_), the primary driving force is the buoyancy‐induced natural convection resulting from temperature gradients. Given the fruit′s placement, the internal air is bifurcated into zones above and below the fruit to calculate the convective exchange separately [15]:

(11) Econvhx=Tsur−TbesthxAfruitΔt0.332Pr13/D2Tsur/Tbesthx1+βt+1g/ϑ12/kairD+0.332Pr13/D2Tsur/Tbesthx1+βt−1gl−y/ϑ12/kairD,

where *g* is the gravitational acceleration (ms^−2^), *β* is the air thermal expansion coefficient (°C^−1^, typically 1/*T*_sur_), *y* is the vertical distance from the refrigerant to the base (meters), *ϑ* is the dynamic diffusivity (m^2^·s^−1^), *D* is the hydraulic diameter (meters), and Pr is the Prandtl number.

**Figure 2:**
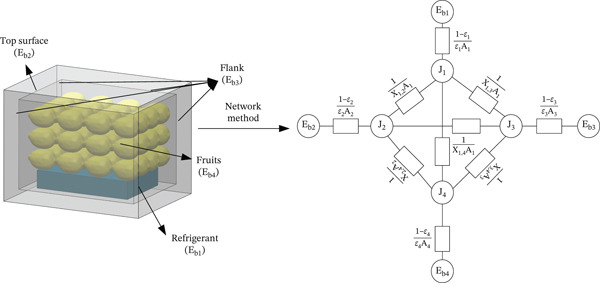
Equivalent thermal radiation network for a four‐surface enclosed cavity, illustrating the coupled heat exchange pathways between the refrigerant, container walls, internal air, and fruit to determine radiative heat gain (*E*_radsurtohx_).

As for *E*_radsurtohx_, it is calculated using the radiation network method for a four‐surface enclosed cavity, as shown in Figure 2. Detailed derivations, including analogies to Kirchhoff′s current law, view factor relationships, and parameter definitions, are referred to [14–16]. The environmental radiative input to the fruit (*E*_radsurtohx_) integrates contributions from the refrigerant, inner walls, and air:

(12) Eradsurtohx= ∑i=13Ei−Ji1−εi/εiAie−x/DΔt,

where *E*_*i*_ is the exponential integral, *ɛ*_*i*_ is the emissivity, and *J*_*i*_ is the effective radiation (W·m^−2^).

Simultaneously, the fruit dissipates heat to the cooler surroundings, represented by the output term (*E*_outhx_) in Equation (1):

(13) Eouthx=E4−J41−ε4/ε4A4e−x/DΔt.

The term *E*_gice_ in Equation (1) represents the sum of the refrigerant′s latent heat of phase change and the sensible heat it undergoes while warming from −5°C to the phase change temperature [14, 15]:

(14) Egice=Micehsf+ciceMiceTs−T0,

where *M*_ice_ is the mass of the phase change refrigerant (kilograms), *h*_sf_ is the latent heat (J·kg^−1^), and *T*_0_ is the initial temperature of the refrigerant (°C).

Combined with Equations (1), (2), (8), and (10)–(14), the storage time of the cold chain container with fruit is as follows:

(15) Δt=10a2ksΔT1+hAΔT2+E1−J11−ε1/ε1A1+E2−J21−ε2/ε2A2+E3−J31−ε3/ε3A3−E4−J41−ε4/ε4A4e−x/D+101010a2ciceMiceTs−Tinitial−a2chxMhxTbest−Tinitialofhx+a2hsfMice+20abksΔT1+hAΔT2+E1−J11−ε1/ε1A1+E2−J21−ε2/ε2A2+E3−J31−ε3/ε3A3−E4−J41−ε4/ε4A4e−x/D+202020abciceMice−abchxMhx+abhsfMice+10b2ksΔT1+hAΔT2+E1−J11−ε1/ε1A1+E2−J21−ε2/ε2A2+E3−J31−ε3/ε3A3−E4−J41−ε4/ε4A4e−x/D−15521101010bVhx+b2ciceMiceTs−Tinitial−b2chxMhxTbest−Tinitialofhx+b2hsfMice,

where *c*_ice_ is the specific heat of refrigerant (J·kg^−1^·°C^−1^).

If the respiratory heat is ignored, Equation (2) is as follows:

(16) ΔEsthx=chxMhxTbesthx−Tinitialhx.

To evaluate the influence of respiratory heat, a simplified model excluding the respiratory heat term (Equation 17) is established for comparison. *E*_inhx_, *E*_outhx_, and *E*_gice_ are the same as Equations (9), (13), and (14), respectively. Therefore, the storage time without respiratory heat is as follows:

(17) Δtwithouthx=Micehsf+ciceMiceTs−Tinitial+chxMhxTinitial−Tbest80.150.54×kXPS+PkXPS+∑Ain−∑Aice−∑Ahx/xkXPS+∑Ahx/1/x/kXPS+Hair/kair+Hhx/khx+Tbest−Tsur+∑Aice/1/Hice/2kice+Hhx/khxTbest−Ts+∑Aice/1/Hice/2kice+x/kXPSTs−Tsur+Tbesthx−TsurAhx0.332Pr13/D2Tsur/Tbesthx1+βt+1gy/ϑ12/kair/D+0.332Pr13/D2Tsur/Tbesthx1+βt−1gl−y/ϑ12/kair/D+E4−J4/1111−ε4/ε4A4−E1−J1/−ε1/ε1A1−E2−J2/−ε2/ε2A2−E3−J3/−ε3/ε3A3e−x/D.

Error is expressed by dividing the difference of theoretical results from their experimental or simulated counterparts by the latter, with the specific calculation formulas given in Equations (18) and (19):

(18) Error=Δt−Δtt0Δtt0×100%,

(19) Error=Δt−Δtt1Δtt1×100%,

where *Δ* *t*_0_ is the storage time of experimental results (minutes) and *Δ* *t*_1_ is the storage time of simulation results (minutes).

### 2.2. Numerical Model

**Figure 3:**
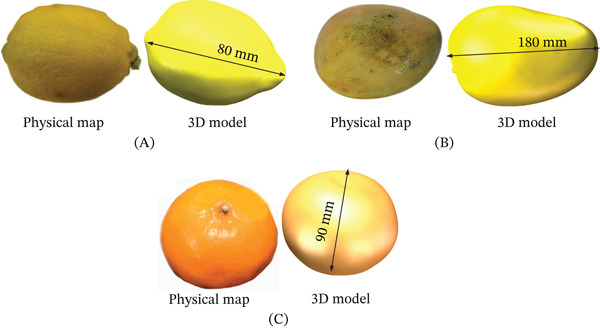
Physical map and 3D model of different fruits: (A) lemon, (B) mango, and (C) orange. The 3D geometries were precisely acquired using a FreeScan EP multifunctional laser scanner to ensure high spatial fidelity in numerical domain meshing.

The fruit models were obtained using a 3D scanner (FreeScan EP, Hangzhou, China). The 3D models of the different fruits are shown in Figure 3. In this work, the investigated fruits are lemon, mango, and orange. The lemons were obtained from Ziyang City, Sichuan Province, and each weighed 70 g, as shown in Figure 3A. The mangoes were obtained from Panzhihua City, Sichuan Province, each weighing 1 kg, as shown in Figure 3B. The oranges were obtained from Nanning City, Guangxi Zhuang Autonomous Region, and each weighed 150 g, as shown in Figure 3C.

**Figure 4:**
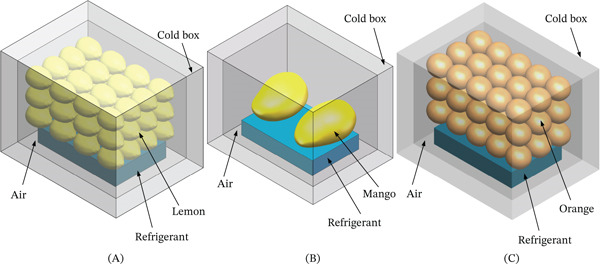
3D model of the cold chain container with different fruits: (A) lemon, (B) mango, and (C) orange. The fruit payload is mathematically modeled as a uniform distribution model (UDM).

The cold chain container′s 3D model is constructed as shown in Figure 4. The cold chain container features interior dimensions of 350 × 250 × 250 mm and a wall thickness of 30 mm. The cold chain container′s material is XPS. The refrigerant is ice, and its size is 275 × 180 × 50 mm. The fruit in the cold chain container is used as a uniform distribution model (UDM) because the contact between the fruits is point [26, 27]. When the contact between the content is point to point, the UDM is applied to calculate the simulation model to reduce calculation time [26].

**Figure 5:**
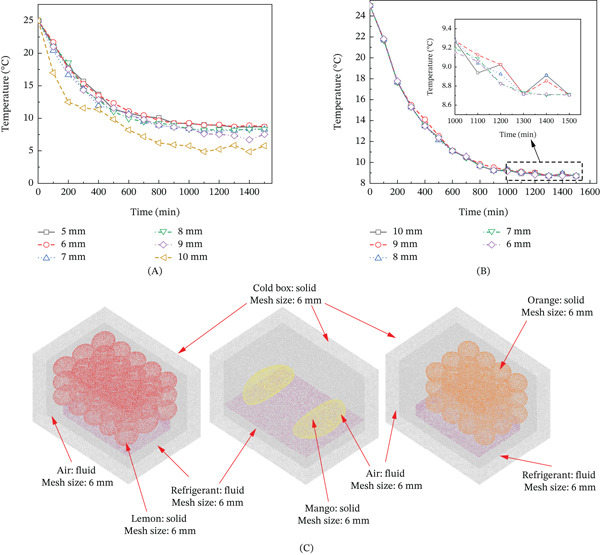
Mesh results of different fruits: (A) the mesh independent of the air part, (B) the influence of mesh size of other parts on the results, and (C) mesh results of different fruits in a cold chain container. (A) Grid independence trajectory for the internal air volume, demonstrating distinct temperature result stabilization once the mesh size is reduced to 6 mm. (B) Sensitivity analysis indicating the negligible influence of structural component mesh sizing on temperature fluctuations below the 6 mm threshold. (C) The finalized 3D hexahedral/tetrahedral mesh configuration applied to the fully loaded cold chain domain, striking an optimal balance between solving time and computational accuracy.

Mesh division is conducted using CFD ICEM. To ensure result accuracy, mesh independence for the air region was verified [28], as shown in Figure 5A. It was determined that reducing the mesh size below 6 mm produced no significant change in results. Therefore, a 6 mm mesh was selected for the air volume. The size of the other parts is also set at 6 mm, thereby reducing the fluctuation of the results, as shown in Figure 5B. The mesh configuration of the cold chain container is presented in Figure 5C.

Temperature exerts no significant influence on the thermal conductivity of XPS [29, 30]. The corresponding material properties for each component are summarized in Table 1.

**Table 1:** Thermophysical properties of the core components of the cold chain container used for theoretical calculation and numerical simulation.

Materials	Thermal conductivity (W·m^−1^ °C^−1^)	Density (kg·m^−3^)	Specific heat (J·kg^−1^ °C^−1^)	Latent heat (J·kg^−1^)
XPS	0.03	33	1500	
Refrigerant	2.22	900	2100	333,000
Air	0.024	1.161	1006	

The numerical simulations were conducted by Fluent 2021 R1, wherein all internal components were treated as continuous media. The model is based on the following principal hypotheses:

1. The cold chain container is treated as nominally sealed, and air leakage is neglected during the calculation.
2. The Boussinesq hypothesis [3, 31] was adopted, and all physical properties were assumed to be constant.
3. The volumetric change in refrigerant during a phase change is neglected.
4. The properties of each part are independent of the temperature change.

The respiratory heat model of fruit is imported into the fruit part of Fluent by using a UDF (user‐defined function). A second‐order upwind scheme was employed for the spatial discretization of both the momentum equations and the volume fraction. An implicit scheme is employed for the discretization of the time term, whereas the phase‐coupled “SIMPLEC” algorithm is used to derive the governing equations for pressure and velocity. Pressure is solved via the “standard” method, with additional setting details provided in Table 2.

**Table 2:** Detailed configuration of the CFD numerical simulation model (Fluent 2021 R1) for the cold chain container loaded with fresh fruits.

Models	Details
Energy equation	Choose energy equation
Viscous	Laminar
Radiation	DO model and off solar load
Gravity	9.81 ms^−2^
Boundary conditions	A no‐slip condition was applied to all walls. The external container wall was assigned a fixed temperature equal to the ambient value, whereas all other interface boundaries were set as coupled
Monitors	Temperature monitoring points were established at four locations: the geometric center of the container, the centers of the top and bottom walls, and the center of the fruit. The sampling interval corresponds to the length of each simulation time step
Time increment	180 s
Maximum iterations	20

## 3. Experiment and Method

### 3.1. Experimental Materials and Equipment

Specifications for the materials and sizes of each part are the same as those of the numerical model. Experimental equipment and their respective applications are listed in Table 3, with the multichannel temperature tester featuring an accuracy of 0.1°C.

**Table 3:** Technical specifications, manufacturers, and specific applications of the experimental testing equipment utilized for thermophysical and biological parameter measurements.

Equipment name	Type	Manufacturers	Application
Multifunctional laser handheld 3D scanner	FreeScan EP	Hangzhou Xianlin Tianyuan Company, China	Construct 3D modeling of fruits
CO_2_ concentration tester	PG‐LG58	GGELE, China	Test the CO_2_ content of fruit produced by respiration
Thermal characteristic analyzer	TEMPOS	METER, United States	Test the thermal conductivity and specific heat capacity of fruit
Multichannel temperature tester	AT4508‐128	Changzhou Anbai Precision Instrument, China	Record the temperature change inside the cold chain container
Environmental chamber with constant temperature and humidity control	ETH‐408‐40‐CP‐AR	Jufu Instrument, China	For simulating the ambient thermal and hygrometric conditions during transit
Refrigerator	MDF‐339‐C	Dalian Sanyang Cold Chain, China	Freeze the refrigerant
Sawing machine	BSM‐400	Tianjin Zhihua Mechanical Electronics, China	Cut XPS plates

### 3.2. Experimental Method of Thermal Properties of Fruit

The SH‐3 sensor (length 35 mm, diameter 1.3 mm) of the thermal characteristic analyzer is used to measure volumetric heat capacity, thermal diffusivity, and thermal resistivity. The SH‐3 sensor is employed in food applications due to its compact form factor. For optimal accuracy, the needles must be parallel with a spacing of 6 mm [32].

The sensor needs to equilibrate for 30 s, and the surrounding temperature drift is reviewed. If the temperature drift is lower than 0.002°C/s, the instrument commences its active heating cycle [33]. During the experiment, the instrument is run for 2 min. The heating process is kept for 30 s, and the temperature measurement is kept for 1.5 min. The temperature measurement is taken at 1‐s intervals.

The temperature change is measured at a point 6 mm from the monitoring needle during heating and the subsequent cooling period, with the results fitted to Equations (20) and (21) via a least‐squares fitting procedure [33].

(20) ΔT=qneedle4πkhxEi−r24Dhxt ΔT=q4πkhxEi−r24Dhxt−th−Ei−r24Dhxt t>th ,

where *Δ* *T* is the temperature increase observed at the measuring needle (°C), *q*_needle_ is the heat input per unit length of the heating probe (W·m^−1^), *r* is the distance between heating and temperature‐sensing probes (meters), *D*_hx_ is the thermal diffusivity of fruit (m^2^·s^−1^), *t* is the time of experiment (seconds), and *t*_h_ is the time of heating (seconds).

The specific heat capacity is calculated as follows:

(21) c=khxρhxDhx,

where *ρ*_hx_ is the density of fruit (kg.m^−3^).

### 3.3. Experimental Method of Respiratory Intensity

We used the widely validated closed‐system method [17, 22] to measure the respiratory intensity of the selected mango, lemon, and orange samples, because its gas accumulation dynamics approximate the restricted gas‐exchange environment inside cold chain containers during transportation. The closed‐system method was selected to measure the respiratory intensity because its gas accumulation dynamics are representative of the actual environment inside the cold chain container. In practical transportation, the container is wrapped with preservative film and tape, which limits free gas exchange. This causes a continuous depletion of O_2_ and accumulation of CO_2_, which in turn progressively inhibits the fruit′s respiration rate through enzymatic feedback. Unlike flow‐through (open) systems that maintain constant gas concentrations, the closed system effectively captures this transient autoinhibition process.

The closed system is housed within a constant‐temperature and constant‐humidity chamber. The temperatures of the chamber are set from 0°C to 50°C (take a temperature point every 5°C). The CO_2_ concentration tester in the closed system is measured every 1 h at each temperature, and the measurement time is 100 h.

### 3.4. Experimental Method of Storage Time of Cold Chain Container

**Figure 6:**
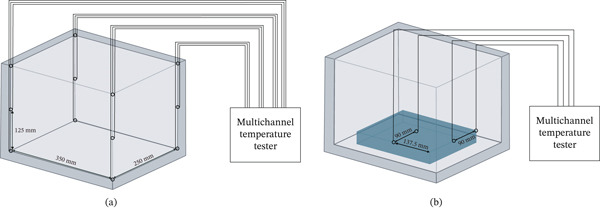
Schematic layout of temperature probes for the container walls and phase change refrigerant. (a) Twelve probes are distributed across the top, middle, and bottom layers of the inner container walls to monitor the 3D spatial temperature gradient. (b) Three auxiliary probes are positioned on the phase change refrigerant to track its melting progression.

This experiment is based on ASTM D3103‐20 [33] and GB/T 33129‐2016 [34]. The recommended transportation temperatures of mango, lemon, and orange all fall within 10°C–14°C [35–37]. Therefore, the storage time of the cold chain container is defined as the duration for which the representative fruit temperature remains at or below 10°C. In the theoretical and numerical analyses, the representative temperature refers to the fruit‐center temperature; in the experiments, it is approximated using surface‐mounted probes placed on representative fruits in each layer. The experimental steps are as follows:

1. Preconditioning: The phase change refrigerant and the fruit samples were preconditioned in separate environments at −10°C and 20°C for 24 h, respectively. Simultaneously, the empty XPS container was placed inside the environmental chamber and maintained at the target ambient testing temperature (e.g., 30°C) for 24 h to reach initial thermal equilibrium.
2. Sensor placement: Multichannel temperature probes were deployed to capture the transient thermal behavior and internal temperature gradients. The specific probe placements were configured separately as follows:

Container walls: Twelve probes were affixed to the four inner corners of the container across three distinct horizontal planes (the bottom, middle, and top layers) to align with the monitoring locations (shown in Figure 6a).

Phase change refrigerant: Four temperature probes were attached to the surface of the phase change refrigerant using adhesive tape to monitor its melting progression (shown in Figure 6b).

**Figure 7:**
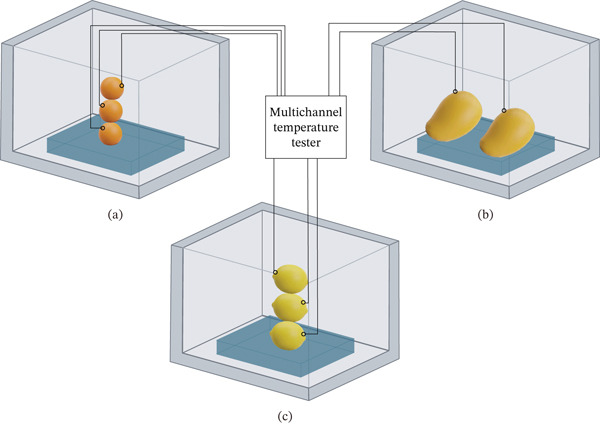
Placement configurations of temperature probes on the surfaces of different fruit samples. To record the transient surface temperature, one probe is attached using adhesive tape to each individual fruit. Consequently, the three‐layer stacked (a) oranges and (c) lemons are equipped with three probes, respectively, whereas the (b) two mangoes are equipped with two probes.

Fruit samples: For the oranges and lemons, which were organized into a three‐layer stacked configuration within the container, three probes were utilized. To ensure representative temperature monitoring across the vertical thermal gradient, one probe was attached to the surface of the fruit positioned at the geometric center of each respective layer (top, middle, and bottom). For the larger mangoes, two probes were attached to distinct surface locations on each individual fruit (as shown in Figure 7).

3. Assembly and sealing: With the environmental chamber preset to the target testing conditions (e.g., 30°C, 50% RH), the preconditioned refrigerant was swiftly placed at the base of the XPS container, followed immediately by the fruit samples. To minimize air infiltration, the structural seams and interfaces of the XPS container were securely sealed using adhesive tape. Subsequently, the entire exterior of the container was tightly wrapped with multiple layers of preservative film.
4. Testing: The fully assembled and tightly sealed cold chain container was immediately transferred into the environmental chamber to commence the transient heat transfer test.
5. Termination: The test was terminated once the temperature readings from the fruit surface probes approached 20°C on the multichannel thermometer. The recorded data were subsequently exported to a computer for postprocessing.
6. Repetition: Following Steps 1–5, the experiment was repeated with the environmental chamber temperature set at 40°C, 50°C, 60°C, and 70°C.

## 4. Results and Discussion

### 4.1. Thermal Properties of Different Fruits

According to Equations (15) and (17), the thermal conductivity and specific heat of the fruits are required to calculate the cold chain container′s storage time. These thermal properties are presented in Table 4.

**Table 4:** Experimentally measured thermophysical properties (density, thermal conductivity, and specific heat capacity) of lemon, mango, and orange for the storage time theoretical model calculation.

Fruit	Density (kg·m^−3^)	Thermal conductivity (W·m^−1^ °C^−1^)	Specific heat (J·kg^−1^ °C^−1^)
Lemon	407.99	0.5884	469.77
Mango	1148.54	0.4073	304.71
Orange	1068.83	0.5492	435.90

### 4.2. Construct a Respiratory Heat Model

**Figure 8:**
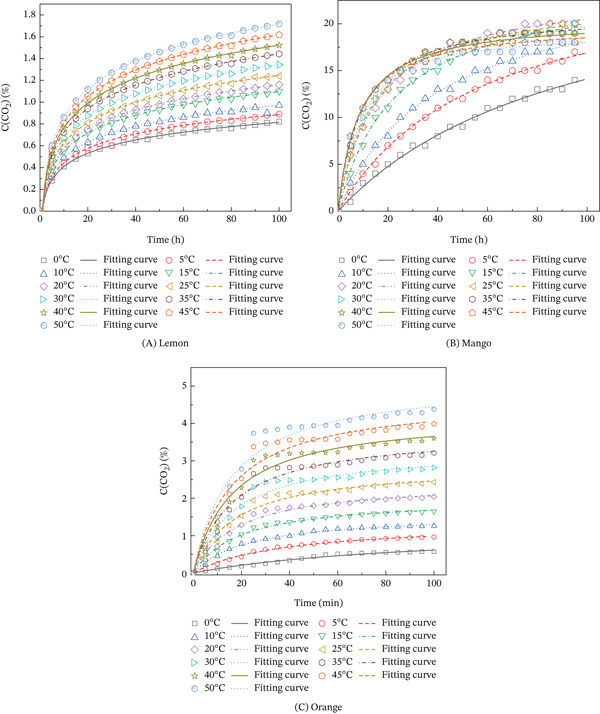
Empirical temporal accumulation of CO_2_ concentration over a 100‐h period in a hermetically closed system under varying ambient temperatures (0°C–50°C) for (A) lemon, (B) mango, and (C) orange. The pronounced, rapid exponential escalation in CO_2_ generation observed in mangoes serves as a clear indicator of their climacteric physiological nature, resulting in a significantly higher metabolic respiratory heat output compared to the relatively linear patterns of the nonclimacteric lemons and oranges.

The respiratory heat models were developed based on CO_2_ concentration data obtained from closed‐system experiments at various temperatures. The CO_2_ concentration change is shown in Figure 8. Compared with lemon and orange, the CO_2_ concentration change of mango is the most significant, because mango is a respiratory climacteric fruit. As for this kind of fruit, the respiratory rate suddenly increases, and then more and more CO_2_ is produced [38].

The varying impact of respiratory heat among the fruits is fundamentally linked to their physiological characteristics. As noted, mango is a climacteric fruit, which experiences a respiratory surge that leads to significantly higher CO_2_ production compared with nonclimacteric fruits like lemon and orange. This surge results in greater metabolic heat generation, explaining why accounting for respiratory heat is particularly critical for climacteric fruits like mango, which exhibit more volatile physiological changes compared with nonclimacteric fruits.

**Figure 9:**
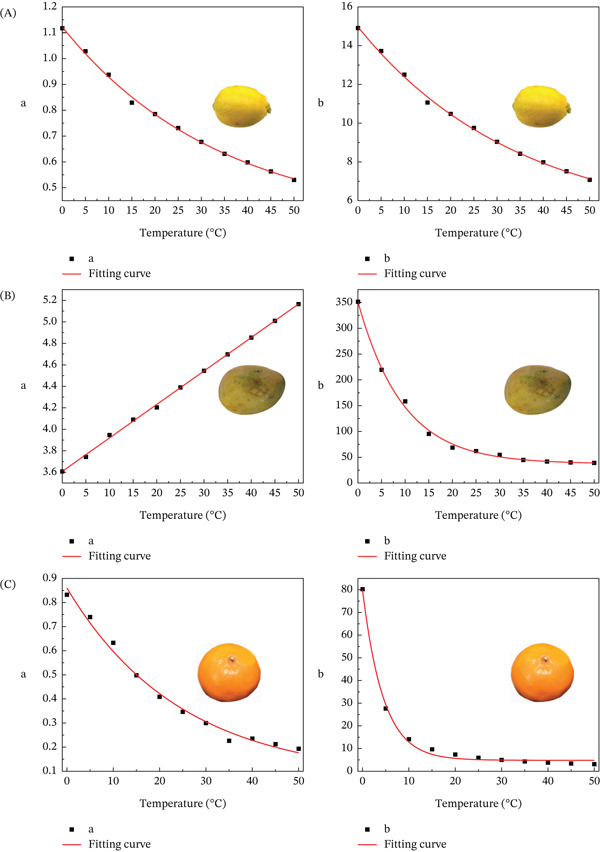
The relationship between parameters *a* and *b* and the temperature of different fruits: the parameter *a*, temperature of lemon on the left of Figure 9A; the parameter *b*, temperature of lemon on the right of Figure 9A; the parameter *a*, temperature of mango on the left of Figure 9B; the parameter *b*, temperature of mango in the right of Figure 9B; the parameter *a*, temperature of orange in the left of Figure 9C; and the parameter *b*, temperature of orange in the left of Figure 9C.

To construct a respiratory heat model with different temperatures, the relationship between the parameters *a* and *b* in Equation (8) and temperature is obtained in Figure 9.

The fitting function of lemon is shown in Equation (22):

(22) alem=0.354520.768050.9976+e−T/34.40305 R2=blem=4.7306410.244260.99808+e−T/34.39082 R2=.

The fitting function of mango is shown in Equation (23):

(23) amango=3.608130.031150.99918+T R2=bmango=37.23621157.12442157.237520.99739+e−T/9.51083+e−T/9.51084 R2=.

The fitting function of orange is shown in Equation (24):

(24) aorange=0.074460.78571+e−T/24.52003borange=4.8227375.079430.99624+e−T/4.48183 R2=.

Substituting Equation (22) into Equation (8), the respiratory heat model of lemon is as follows:

(25) Qrlemon=0.552282××Vhx4.7306410.24426+e−T/34.39082Δt60.354520.76805+e−T/34.40305Δt+4.7306410.24426+e−T/34.390822.

Substituting Equation (23) into Equation (8), the respiratory heat model of mango is as follows:

(26) Qrmango=0.552282××Vhx37.23621157.12442157.23752+e−T/9.51083+e−T/9.51084Δt63.608130.03115+TΔt+37.23621157.12442157.23752+e−T/9.51083+e−T/9.510842.

Substituting Equation (24) into Equation (8), the respiratory heat model of orange is as follows:

(27) Qrorange=0.552282××Vhx4.8227375.07943+e−T/4.48183Δt60.074460.78571+e−T/24.52003Δt+4.8227375.07943+e−T/4.481832.

We discuss the physical significance of the fitted parameters *a* and *b* to further elucidate the biological implications of the respiratory model beyond its statistical fitting performance (*R* ^2^). The term 1/*b* represents the initial respiration intensity at the start of storage (*Δ* *t*⟶0) based on the derivation in Equation (7). As observed in Figure 9, the decrease in *b* with rising temperature reflects the acceleration of enzyme‐catalyzed reactions, leading to a more vigorous initial metabolism. On the other hand, 1/*a* represents the asymptotic saturation level of CO_2_ concentration in the closed test system. This mechanistic understanding explains why the respiratory heat *Q*_r_ (Equation 8) accumulates more rapidly at higher ambient temperatures, thereby fundamentally shortening the storage longevity (*Δ* *t*).

### 4.3. Accuracy of the Theoretical Model of Storage Time With Different Fruits

**Figure 10:**
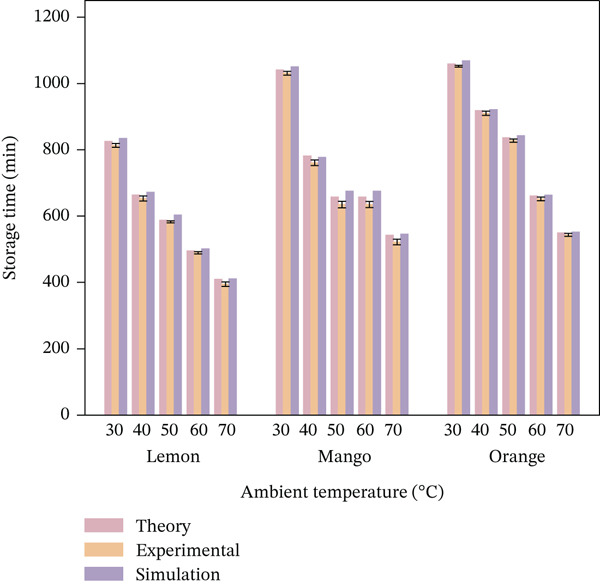
Comparison of the valid storage time of cold chain containers loaded with lemon, mango, and orange under different ambient temperatures, obtained from the proposed respiratory heat‐coupled theoretical model, CFD numerical simulation, and experimental test, verifying the good agreement and high prediction accuracy of the theoretical model.

By substituting Equations (25)–(27) into Equation (15), the theoretical model incorporating respiratory heat is obtained for different fruits. The storage time was identified when the representative fruit temperature reached 10°C. For the theoretical and numerical models, the representative temperature corresponds to the fruit‐center temperature, whereas for the experiments, it was estimated using surface‐mounted probes on representative fruits. Across varying ambient temperatures, the theoretical results were benchmarked against both experimental data and simulation outcomes, as shown in Figure 10. The errors are calculated by Equations (17) and (18).

As shown in Figure 10, the maximum discrepancy between the theoretical model and experimental results is only 3.90%, confirming the model′s high predictive accuracy. This minor deviation primarily stems from the difference between the experimental conditions and the model assumptions. In the theoretical and numerical models, the cold chain container is assumed to be an ideal, perfectly sealed system. However, in the actual experiments, despite the rigorous physical sealing measures applied (i.e., tape and preservative film), the container is not perfectly sealed due to the inherent microscopic permeability of the materials and minute interfacial gaps. This unavoidable microleakage allows continuous air infiltration from the external hot environment into the container. Such infiltration introduces not only sensible heat but also latent heat when the external water vapor condenses on the cold surface of the phase change refrigerant. This unquantified advective heat load accelerates the melting process of the refrigerant, which explains why the actual experimental storage times are generally slightly shorter than the theoretical predictions.

The discrepancy between the simulation and experimental results is due to the cold chain container not being sealed in experiments and the surface of the fruits not being considered in the simulation [37]. A maximum discrepancy of 6.30% was observed between the simulation and experimental results. This result indicated that the simulation model can also predict the cold chain container′s storage time. In comparison to the predictive accuracy of the simulations, the theoretical model exhibited a lower error, which demonstrates the higher accuracy of the theoretical model over the simulation for storage time prediction.

The theoretical model and simulation results exhibit excellent agreement, with a maximum discrepancy of only 2.59%. The result of the error is due to the difference in solution mode [14]. To evaluate the advantages of the theoretical model, a comparative analysis was conducted between it and the simulation model in terms of calculation methodology, time, and results. The computer configurations of the theoretical model and simulation model are both Intel Xeon CPU E5‐1620 V3, 3.5 GHz, 32 GB RAM. The comparison results are shown in Table 5.

**Table 5:** Performance comparison between the proposed respiratory heat‐coupled theoretical model and the CFD numerical simulation model in terms of calculation method, time consumption, and prediction accuracy.

Aspects	Theoretical model	Simulation model
Calculation method	Directly	Iteratively using the separation method
Calculation time	Within 10 s (by MATLAB software)	60–400 min (it is affected by the amount of refrigerant and computer configuration)
Calculation results	The result is more accurate than the simulation model results	The result is approximate. The storage time and temperature distribution are both obtained

As for the theoretical model, the solution mode is a direct calculation method, and the result is exact [14, 15]. As for the simulation model, the solution mode is a separate iterative calculation method, and the result is approximate [14, 15]. Compared with the simulation model, the theoretical model can reduce time and predict the experimental results well. The theoretical model is not impacted by the computer hardware specification.

**Figure 11:**
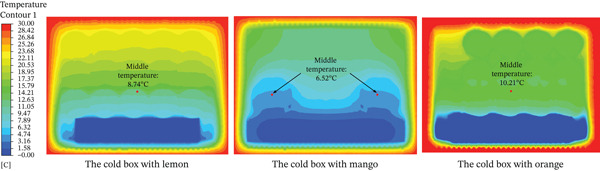
Temperature distribution characteristics inside the cold chain containers loaded with lemon, mango, and orange under 30°C ambient temperature, showing the temperature difference of the fruit center at the end of the valid storage period.

The temperature distributions of different fruits are shown in Figure 11 during the storage time under 30°C ambient temperature. The middle temperature of lemon is 8.74°C, the middle temperature of mango is 6.52°C, and the middle temperature of orange is 10.21°C.

### 4.4. Influence of Respiratory Heat on the Storage Time With Different Fruits

**Figure 12:**
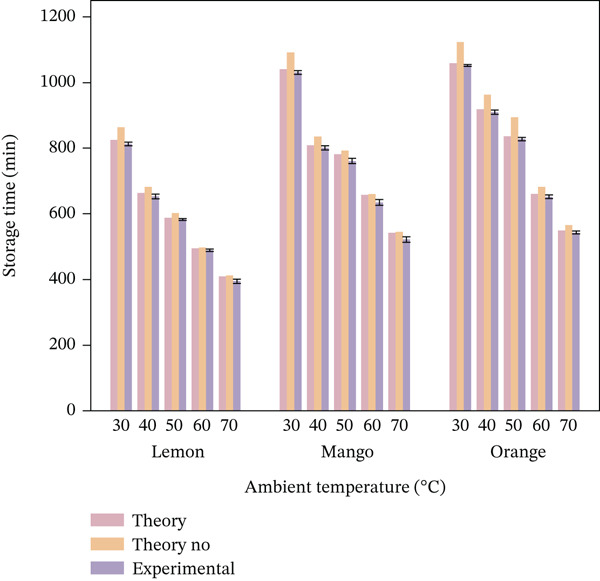
Comparison of the valid storage time between the theoretical model without respiratory heat and experimental test for cold chain containers loaded with lemon, mango, and orange, quantifying the increased prediction deviation caused by ignoring fruit respiratory heat.

To obtain the impact of respiratory heat on the storage time with different fruits, the theoretical model of storage time without respiratory heat (Equation 17) is used to compare with experimental results. The results of the model excluding respiratory heat are shown in Figure 12. The errors are calculated by Equation (18).

As shown in Figure 12, when respiratory heat is excluded from the theoretical model, the maximum prediction error increases to 8.00%. Comparing this with the fully coupled model demonstrates that incorporating the fruit respiratory heat significantly enhances predictive accuracy. Furthermore, Figure 12 reveals that the discrepancy between the models with and without respiration narrows progressively as the ambient temperature increases. This indicates that at extremely high ambient temperatures (e.g., 60°C and 70°C), the thermal properties of fruits have a greater impact on storage time than respiratory heat. Therefore, constructing a respiratory heat model of fruits can improve the predictive performance of the theoretical model.

### 4.5. Practical Implications

The proposed theoretical model provides a quantitative decision‐making tool for cold chain logistics management. Primarily, the model guides mixed loading strategies to ensure thermal compatibility and prevent spoilage in multiproduct shipments by accounting for the distinct respiratory heat profiles of various fruits. For instance, when transporting climacteric fruits with high metabolic heat generation, such as mangoes, alongside nonclimacteric fruits like lemons, the model can quantitatively evaluate the aggravated internal heat load (*Q*_*r*_). Furthermore, it enables the precise determination of the phase change refrigerant quantity (*M*_ice_) required for specific storage durations (*Δ* *t*), optimizing the balance between cooling capacity and payload efficiency. By calculating exact cooling needs for heat gain and respiration, the model avoids overpacking refrigerants that otherwise reduce valuable cargo space. Finally, given its exceptionally high computational efficiency (solving within seconds compared with hours required for CFD simulations), the model can be readily integrated into transport route planning systems to dynamically adjust logistics schedules based on real‐time predictions of the remaining storage window.

## 5. Conclusion

In this study, the relationship between the respiratory heat of fruits and cold chain container storage time is analyzed. A theoretical model for predicting storage time was derived by integrating conduction, convection, radiation, and respiratory heat transfer, taking lemon, mango, and orange as examples.

The shape factor is applied to establish the conduction heat transfer. The gravity and temperature differences are coupled to calculate the convection heat transfer. The network method is applied to constitute the radiation heat transfer. The respiratory heat models of different fruits at different temperatures are constructed. The theoretical model coupling heat transfer and the respiratory heat model is compared with simulation and experimental results. A maximum discrepancy of 3.90% was observed between the simulation and experimental results, indicating that the theoretical model can predict the experiment. A maximum discrepancy of 6.30% was observed for simulation results relative to experimental data, which demonstrates that the theoretical model is more accurate than simulation in predicting storage time. The impact of respiratory heat on storage time is analyzed by comparing a respiratory heat–excluded theoretical model with experimental results. A maximum error of 8.00% was observed, which indicates that fruit respiratory heat affects the storage time. The theoretical model incorporating respiratory heat provides more accurate predictions of the experimental data than the model excluding it.

In this study, the theoretical model coupling conduction–convection–radiation–respiratory heat transfer can be used to calculate a 3D closed environment with different contents with respiratory.

## Author Contributions

Liying Duan: conceptualization, methodology formulation, theoretical model development, formal analysis, writing—original draft preparation, and visualization; Haozhe Liu: numerical simulation implementation, data curation, experimental investigation, and writing—review and editing; Zhiqiang Fu: conceptualization, overall project supervision, provision of experimental resources, methodology validation, and writing—review and editing; Liqiang Huang: execution of experimental tests, validation of computational results, critical review of the manuscript, and contribution to the data interpretation; Yan Wang: methodological supervision, assistance with data analysis, visualization support, and manuscript proofreading.

## Funding

This work was supported by the Doctoral Innovation Fund Project of Tangshan University (Grant No. BC202104), Key Laboratory of Agro‐Products Processing and Storage, Ministry of Agriculture and Rural Affairs (Grant No. S2022KFKT‐14).

## Disclosure

All authors have read and approved the final version of the manuscript and agree to be accountable for all aspects of the work in ensuring that questions related to the accuracy or integrity of any part of the article are appropriately investigated and resolved.

## Conflicts of Interest

The authors declare no conflicts of interest.

## Data Availability

Upon reasonable request, the data supporting this study′s findings can be obtained from the corresponding author.
